# Effects of a maintenance period on ambulatory blood pressure and morning blood pressure surge in young normotensives post isometric training

**DOI:** 10.3389/fphys.2024.1405230

**Published:** 2024-08-15

**Authors:** A. W. Baross, B. A. Baxter, B. H. Wright, A. D. Kay

**Affiliations:** ^1^ Sport and Exercise Science, University of Northampton, Northampton, United Kingdom; ^2^ Health and Life Sciences, Oxford Brookes University, Oxford, United Kingdom

**Keywords:** maintenance, ambulatory blood pressure, isometric, resistance, training

## Abstract

Isometric resistance training (IRT) has emerged as an efficacious therapeutic intervention to reduce ambulatory blood pressure (BP), and BP diurnal variability. However, once the required decreases in BP have been achieved the efficacy of implementing a reduced maintenance dose is not understood. Therefore, the purpose of this study was to determine the effects of an 8-week maintenance period (8-week) following the cessation of the prescribed 8-week IRT in young normotensives. Twenty-two recreationally active, not resistance trained, normotensive (24-h ambulatory SBP, ≥130 mmHg) young adults were randomly assigned to a training-maintenance [TG-MT; *n* = 13 (female = 5); age 21 ± 2 years] or a non-training control [CON; *n* = 9 (female = 4); age 23 ± 3 years] group. Ambulatory BP, morning BP surge (MBPS) and average real variability (ARV) were measured prior to, after 8 weeks of bilateral leg IRT (4x2-minute contractions at 20% MVC with 2-min rest periods, 3 days/week) and following an 8-week (once per week) maintenance period. On completion of the maintenance period the significant reductions seen following the IRT were maintained within the TG-MT group in 24-h ambulatory SBP (6 ± 4 mmHg, *p* < 0.001), daytime (5 ± 5 mmHg, *p* = 0.002), MBPS (7 ± 10 mmHg, *p* = 0.019) and 24-h SBP ARV (2.03 ± 1.44 mmHg, *p* = 0.001), daytime SBP ARV (2.04 ± 1.78 mmHg, *p* = 0.003). These results show that reductions in ambulatory BP (24-h SBP and daytime SBP), in addition to BP diurnal variations (MBPS, 24-h SBP ARV and daytime SBP AVR) are maintained following an 8-week maintenance dose in young adults and add further weight to the growing body of evidence promoting IRT as an efficacious therapeutic exercise intervention to prevent or reduce BP.

## Introduction

At present there is estimated to be over 1.6 billion individuals worldwide with hypertension, a main risk factor for coronary heart disease (CHD), cardiovascular disease (CVD) and stroke (WHO, 2013; WHO, 2016; Lopes et al., 2021), which are responsible for over 8.5 million deaths globally each year (Zhou et al., 2021). Interestingly, although more prevalent in older adults, the incidence of hypertension is increasing in active younger populations (De Venecia et al., 2016; Hamrahain and Falkner, 2022). As young adults tend to have less health awareness than older individuals, early identification of “at risk” individuals accompanied by preventative therapeutic exercise treatments could prevent the development of hypertension in later years.

Day-to-day variation in 24 hour (24-h) ambulatory blood pressure (BP), in particular night-time ambulatory BP, are also strongly associated with these cardiovascular risk factors, more so than resting BP (Whelton et al., 2018; Mancia et al., 2023; Staplin et al., 2023). In addition, morning blood pressure surge (MBPS), the normal surge in BP seen upon waking, as a result of the drop in nocturnal BP, has been reported to have a close association with end organ damage and is considered to be a disabling factor for atherosclerotic plaques (White, 2001; Kario, 2005; Kario, 2010). Therefore, research examining the impact of interventions on hypertension need to consider measuring ambulatory BP and the morning surge in blood flow in addition to resting BP.

Isometric resistance training (IRT) has emerged as an efficacious therapeutic intervention to reduce ambulatory BP when performed up to 10 weeks, when performing handgrip (Somani et al., 2017) or lower body (wall squat, Taylor et al., 2019; knee extension; Baross et al., 2022b) exercises. Furthermore, IRT is considered to be one of the leading non-pharmacological therapeutic interventions for the treatment of hypertension (Whelton and Carey, 2017) and has been included in the most recently published Australian position stand on Exercise and Hypertension (Sharman et al., 2019). Research examining IRT has demonstrated reductions in 24-h, daytime, and night-time ambulatory BP (Somani et al., 2017; Taylor et al., 2019) in addition to decreases in MBPS (Baross et al., 2022a) and average real variability (ARV; Baross et al., 2022b); a measure of the average absolute changes in BP between consecutive readings over a set period (24-h, daytime or night-time). Therefore, IRT may be considered to be an efficacious non-pharmacological strategy to reduce BP that does not require specialist equipment, however for an intervention to be effective, patients must adhere to the recommendations.

Despite the benefits of physical activity interventions, including IRT, adherence and compliance to prescribed exercise programmes are unfortunately, low (Bennie et al., 2019; Lopes et al., 2021). Leijon et al. (2011) and Hennein et al. (2018) reported that the increase in the prevalence of hypertension is in part due to a lack of patient adherence to the prescribed pharmacological or therapeutic physical activity interventions. Indeed, regular exercise adherence tends to decline over time (Bennie et al., 2019), with only 50% of individuals continuing with regular exercise within the first 12 months following the completion of the prescribed therapeutic treatment, with a lack of perceived time cited as one of the main barriers (Gee et al., 2012). To minimise the demands of an exercise intervention to potentially enhance adherence, the volume of training could be decreased once the initial training-induced adaptations have been elicited (i.e., a maintenance dose). To the author’s knowledge, only Cohen et al. (2023) has examined the effects of a maintenance dose (one session of 4 × 2-min contractions a week) once the desired reductions in BP have been achieved. The most commonly used IRT protocol employs a thrice-weekly training programme consisting of 4 × 2-min isometric contractions, separated by 2-min of recovery with limited research into dose response effects (Smart et al., 2019). Therefore, once the required decreases in BP (resting or ambulatory) have been achieved, the efficacy of implementing a maintenance dose are not fully understood, despite the possible benefits of a reduced training frequency on individual’s long-term adherence and compliance.

Therefore, to further determine the efficacy of IRT as an alternative therapeutic approach to lower ambulatory BP it is important to quantify the effects of a maintenance dose following a successful IRT intervention. Therefore, the aim of the present study was to determine the effects of a maintenance period (8 weeks) following the cessation of the originally prescribed IRT (8 weeks) intervention on ambulatory BP, MBPS and ARV in young normotensive males and females.

## Materials and methods

### Participants

Twenty-two moderately active (IPAQ) but not resistance trained, normotensive (24-h ambulatory SBP, ≥130 mmHg) individuals (Females; *n* = 9; age 21 ± 4 years; Males; *n* = 13; age 22 ± 4 years) were recruited and randomly assigned (Urbaniak and Plous, (2013). Research Randomizer (Version 4.0) [Computer software]) to either a training-maintenance (TG-MT; *n* = 13 [female = 5]; age 21 ± 2 years, height 169 ± 9 cm, mass 73 ± 18 kg) or a non-training control (CON; *n* = 9 [female = 4]; age 23 ± 3 years, height 170 ± 10 cm, mass 74 ± 8 kg) group. As previous research (Somani et al., 2017) has noted no significant difference in BP changes between males and females following IRT, mixed-sex groups were used. Participants undertook three IRT sessions per week, did not engage in smoking or vaping (Bolinder and de Faire, 1998; Crippa et al., 2018) and were not prescribed medication. Following the University of Northampton’s institutional ethical approval, all participants received a detailed information sheet explaining the experimental protocol and potential risks involved, then completed and signed an informed consent form and pre-test medical questionnaire; all procedures were conducted in line with the Declaration of Helsinki.

An initial session was conducted prior to baseline data collection to familiarise participants with the measures and protocols. Participants then undertook 8 weeks of supervised IRT, performing bilateral isometric leg extensions with post-intervention testing (mid-point) undertaken following the final training session. The maintenance period commenced the week following the final training session and continued for 8 weeks, with participants undertaking one IRT session week. Post maintenance measures (end-point) were recorded following the final week of the 8-week maintenance phase. All data collection sessions were undertaken at the same time of day (±2 h) by the same individual in a temperature-controlled environment (20ºC–23°C) at least 2 h post prandial and within 48 h of the final training session with participants instructed to refrain for training during this time. Participants were asked to avoid over-the-counter medication, vigorous exercise, or caffeine and alcohol for 12 h and 24 h, respectively prior to data collection. All data collection measures in females were taken in the early follicular phase (days 1–7 of the menstrual cycle; *n* = 2) or during placebo pill ingestion for females taking oral contraceptives (*n* = 8; Tsai et al., 2003; Somani et al., 2017). All procedures were conducted with the intent to treat.

## Protocol

### Ambulatory blood pressure

A portable BP monitor (P.M.S. Instruments Ltd. Maidenhead, Berks, United Kingdom) was used to measure 24-h, daytime and night-time ambulatory BP. Night-time measures (recorded every 60 min) were defined as the time participants went to bed until they awoke and daytime measures (recorded every 30 min) as the time participants rose until they retired to bed (Amici et al., 2009; Kario, 2010; Verdecchia et al., 2012). Data for 24-h ambulatory BP recordings were discarded when there was a missing hour of data or ≤86% valid measures were present (Gosse et al., 2004; Hernández-del Rey et al., 2007). To standardise the BP measures, participants reported the activities undertaken during the baseline ambulatory BP monitoring period, including their sleep patterns, and were subsequently asked to undertake similar activities and sleep periods for the ambulatory BP measure at post-intervention and maintenance data collection.

### Morning blood pressure surge and average real variability calculations

As previously reported (Baross et al., 2022b), MBPS was calculated as the mean of the four systolic BP (SBP) readings in the 2-h period just after waking minus the mean of the two readings centred around the lowest nocturnal SBP reading but not adjusted for 24-h ambulatory BP (Kario, 2005; Amici et al., 2009; Verdecchia et al., 2012). ARV of ambulatory (24-h, daytime and night-time) SBP and diastolic BP (DBP), a measure of BP variability, was calculated as previously reported (Boardman et al., 2017). Participant baseline data including mean values for MBPS are presented in Table 1.

**TABLE 1:** Participant baseline demographic and ambulatory data.

	TG-MT group (n = 13)	CON group (n = 9)
Age (yrs)	21 ± 2	23 ± 3
Height (cm)	169 ± 9	170 ± 10
Body Mass (kg)	73 ± 18	74 ± 8
Resting Heart Rate	69 ± 10	69 ± 9
*Ambulatory BP*		
24-h SBP (mmHg)	120 ± 6	121 ± 5
24-h DBP (mmHg)	64 ± 6	64 ± 6
Daytime SBP (mmHg)	123 ± 5	125 ± 6
Daytime DBP (mmHg)	71 ± 6	66 ± 8
Night-time SBP (mmHg)	108 ± 7	105 ± 3
Night-time DBP (mmHg)	55 ± 8	56 ± 7
Morning SBP (mmHg)	122 ± 7	120 ± 4
Lowest Night-time SBP (mmHg)	96 ± 8	97 ± 4
MBPS (mmHg)	26 ± 7	23 ± 6

Values are means ± SD. TG-MT, maintenance group; CON, control group; SBP, systolic blood pressure; DBP, diastolic blood pressure; MAP, mean arterial pressure; MBPS, morning blood pressure surge.

### Isometric resistance training

Participants completed 16 weeks of supervised, on-site IRT using an isokinetic dynamometer (Biodex Medical Systems Inc. New York, United States). During the initial 8 weeks, participants undertook IRT three times per week (separated by at least 24 h), and during the second 8 weeks a maintenance phase IRT was undertaken once per week. Each training session consisted of 4 × 2-min isometric contractions at 20% of maximal voluntary isometric strength (MVC), with 2-min rest periods between contractions. MVC was assessed prior to the start of the programme and reassessed at the end of weeks 2, 4 and 6 of both the training intervention and maintenance phase, determined as the greatest value following three 2–4 s maximum isometric bilateral knee extensions (Baross et al., 2013). Additionally, dietary, nutritional and exercise changes were monitored and recorded throughout the 8-week intervention and maintenance periods using personal logs.

### Statistical analysis

All data satisfied parametric assumptions (Field, 2000) and are presented as mean ± SD; statistical significance was set at *p* < 0.05. Statistical analyses were performed using IBM SPSS Statistics v.26 software (SPSS Inc., Chicago, Illinois, United States). Two-way mixed-model ANOVAs (time [baseline, post-intervention, post-maintenance] × group [TG-MT, CON]) were used to examine within- and between-subject effects for ambulatory BP (mean 24-h, daytime, night-time and diurnal variation), MBPS, and ARV at baseline compared to post-intervention (mid-point) and the maintenance period (endpoint). Post-hoc analyses (Bonferroni) were used to determine the specific location of any significant differences.

The day-to-day reliability of the ambulatory BP measures has previously been reported (Baross et al., 2022b; Baross et al., 2022a) with intraclass correlation coefficient (ICC) values of 0.73, 0.71 and 0.81 and the coefficients of variation (expressed as a percentage of the mean) of 2.5%, 2.3%, and 2.6%, for 24-h SBP, daytime SBP and night-time SBP, respectively.

### Sample size

*A-priori* sample size calculation using G*Power (v.3.1 Düsseldorf, Germany) was used to determine how many participants were required to reach statistical power using alpha = 0.05 and power = 0.80; effect sizes (24 h SBP [*d* = 1.33], Daytime SBP [*d* = 1.38], MBPS [*d* = 1.40]) from similar studies (Somani et al., 2017; Baross et al., 2022a) revealed that a total of 20 participants were needed. To account for 20% attrition, a minimum of 24 participants were initially recruited.

## Results

Participants completed ≥95% of the twenty-four training sessions over the 8-week IRT programme. There was no significant difference (*p* > 0.05 in all cases) in baseline data between the TG-MT and CON groups for age, height, body mass, and all BP measures (Table 1). No changes in the participants’ exercise routine or diet were reported throughout the 16-week training intervention and maintenance period. Body mass (kg) was maintained throughout the 16-week training and maintenance period (TG-MT pre-training = 73.4 ± 18.4, post-training = 73.3 ± 18.0, post-maintenance = 72.9 ± 17.1; CON pre-training = 73.7 ± 8.4, post-training = 73.8 ± 8.5, post-maintenance = 73.4 ± 8.4; *p* > 0.05 in all cases). There were no reported adverse effects of the training intervention or the data collection sessions.

### Effects of IRT on ambulatory blood pressure, MBPS and ARV

Following the 8-week IRT programme there were significant reductions in 24-h ambulatory SBP (−7 ± 5 mmHg, *p* = 0.001) and daytime SBP (−4 ± 5 mmHg, *p* = 0.034) within the TG-MT group. In addition, MBPS significantly reduced (−8 ± 9 mmHg, *p* = 0.008) over time, as did the TG-MT group’s 24-h and daytime SBP ambulatory ARV (−2.10 ± 1.71 mmHg, *p* = 0.033; −2.34 ± 2.49 mmHg, *p =* 0.002, respectively). However, there was no significant change in night-time SPB (−2 ± 5 mmHg, *p* = 0.685) or night-time SBP ARV (−0.76 ± 4.00 mmHg, *p* = 1.000). Additionally, over time there were no observed changes in ambulatory DBP (24-h, 0 ± 6 mmHg; daytime, −2 ± 7 mmHg; night-time, 1 ± 7 mmHg) within the TG-MT group, (*p* > 0.05 in all cases), nor were there any changes in 24-h, daytime and night-time DBP ARV (*p* > 0.05 in all cases). Furthermore, there were no significant differences in the CON group’s ambulatory BP, MBPS or ARV (24-h, daytime, night-time) over the same period (*p* > 0.05 in all cases, see Figures 2A, B; Table 2).

**TABLE 2:** Ambulatory SBP ARV results at baseline, post 8-week IRT and post 8-week maintenance period TRG-MT Control.

Ambulatory SBP ARV (mmHg	Baseline	Post 8-week IRT	Post 8-week maintenance	Baseline	Post 8-week IRT	Post 8-week maintenance
24-h	10.29 ± 1.72	8.20 ± 1.51*	8.26 ± 1.05**	10.12 ± 1.78	10.45 ± 1.80	10.39 ± 2.21
Daytime	10.77 ± 2.07	8.45 ± 2.07**	8.72 ± 1.16**	10.45 ± 2.40	10.04 ± 1.79	10.67 ± 2.56
Night-time	8.92 ± 2.84	8.17 ± 3.17	8.27 ± 1.68	8.11 ± 1.97	8.24 ± 2.78	8.61 ± 1.94

Data are presented as mean ± SD. (TRG-MT, group *n* = 13; Control group *n* = 12). SBP, systolic blood pressure; ARV, average real variability. *p* values represent changes within groups over time compared to baseline measures. **p-value < 0.01, *p-value < 0.05.

### Effects of maintenance period on ambulatory blood pressure, ARV and MBPS

**FIGURE 1:**
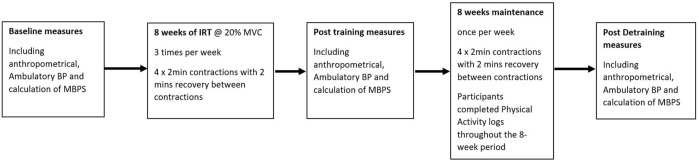
Study flow diagram illustrating the Isometric resistance training (IRT) and maintenance periods and the points of blood pressure measures.

**FIGURE 2:**
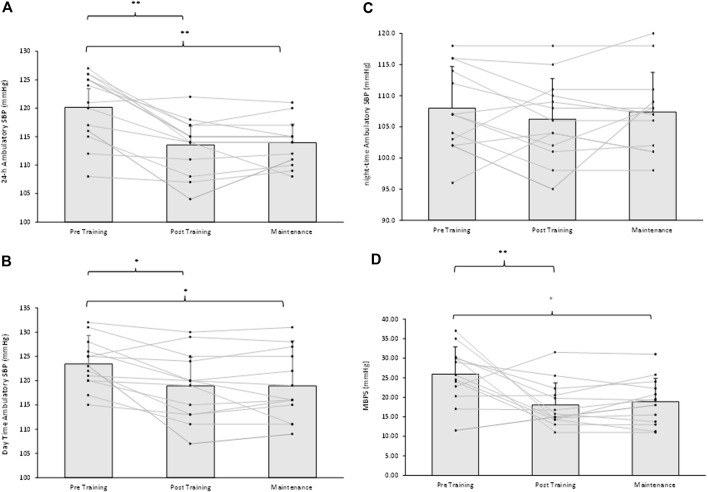
Effects of 8 weeks of isometric resistance training (IRT) followed by 8 weeks maintenance period for individual participants and mean data on **(A)**, 24-h **(B)**, Daytime **(C)**, Night-time ambulatory systolic blood pressure (SBP) and **(D)**, morning blood pressure surge (MBPS) for isometric training-maintenance (TRG-MT) group. *p-value < 0.05, **p-value < 0.01.

**FIGURE 3:**
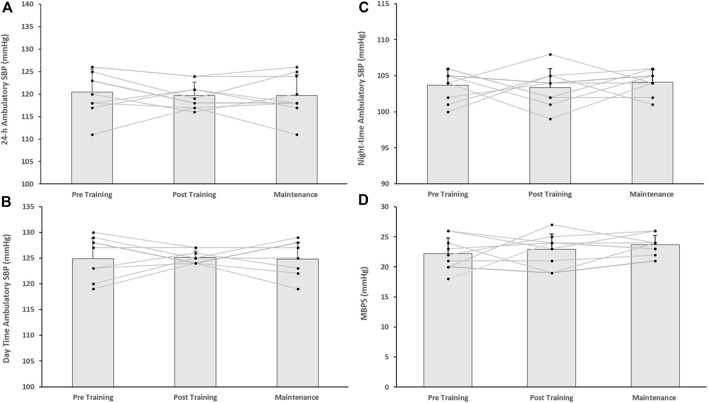
Effects of 8 weeks of isometric resistance training (IRT) followed by 8 weeks maintenance period for individual participants and mean data on **(A)**, 24-h **(B)**, Daytime **(C)**, Night-time ambulatory systolic blood pressure (SBP) and **(D)**, morning blood pressure surge (MBPS) for the control (CON) group.

On completion of the 8-week maintenance period, ambulatory SBP, ARV (both, 24-h, daytime) and MBPS did not change from post training levels (24-h; 114 ± 5 to 114 ± 4 mmHg; daytime; 119 ± 7 to 119 ± 7 mmHg; ARV; 24-h; 8.20 ± 1.51 to 8.26 ± 1.07 mmHg; daytime; 8.43 ± 2.07 to 8.72 ± 1.16 mmHg; MPBS; 18 ± 6 to 19 ± 6 mmHg, respectively, *p* > 0.05 in all cases). As a consequence, significant reductions were maintained from baseline measures within the TG-MT group in 24-h ambulatory SBP (−6 ± 4 mmHg, *p* < 0.001), daytime (−5 ± 5 mmHg, *p* = 0.002), MBPS (−7 ± 10 mmHg, *p* = 0.019) and 24-h SBP ARV (−2.03 ± 1.44 mmHg, *p* = 0.001), daytime SBP ARV (−2.04 ± 1.78 mmHg, *p* = 0.003, see Figures 1–3; Table 2). There were no significant changes in night-time SPB (−1 ± 6 mmHg, *p* = 0.940) or night-time SBP ARV (−0.66 ± 2.80 mmHg, *p* = 1.000). Additionally, over time within the TG-MT group there were no significant changes in ambulatory DBP (24-h, 0.0 ± 5 mmHg; daytime, 2 ± 7; night-time, −2 ± 8, *p* > 0.05 in all cases) or DBP ARV (24-h, 0.23 ± 1.90 mmHg; daytime, 0.10 ± 1.93; night-time, −0.12 ± 3.34., *p* > 0.05 in all cases).

### Between group ambulatory blood pressure, MBPS and ARV analysis

**FIGURE 4:**
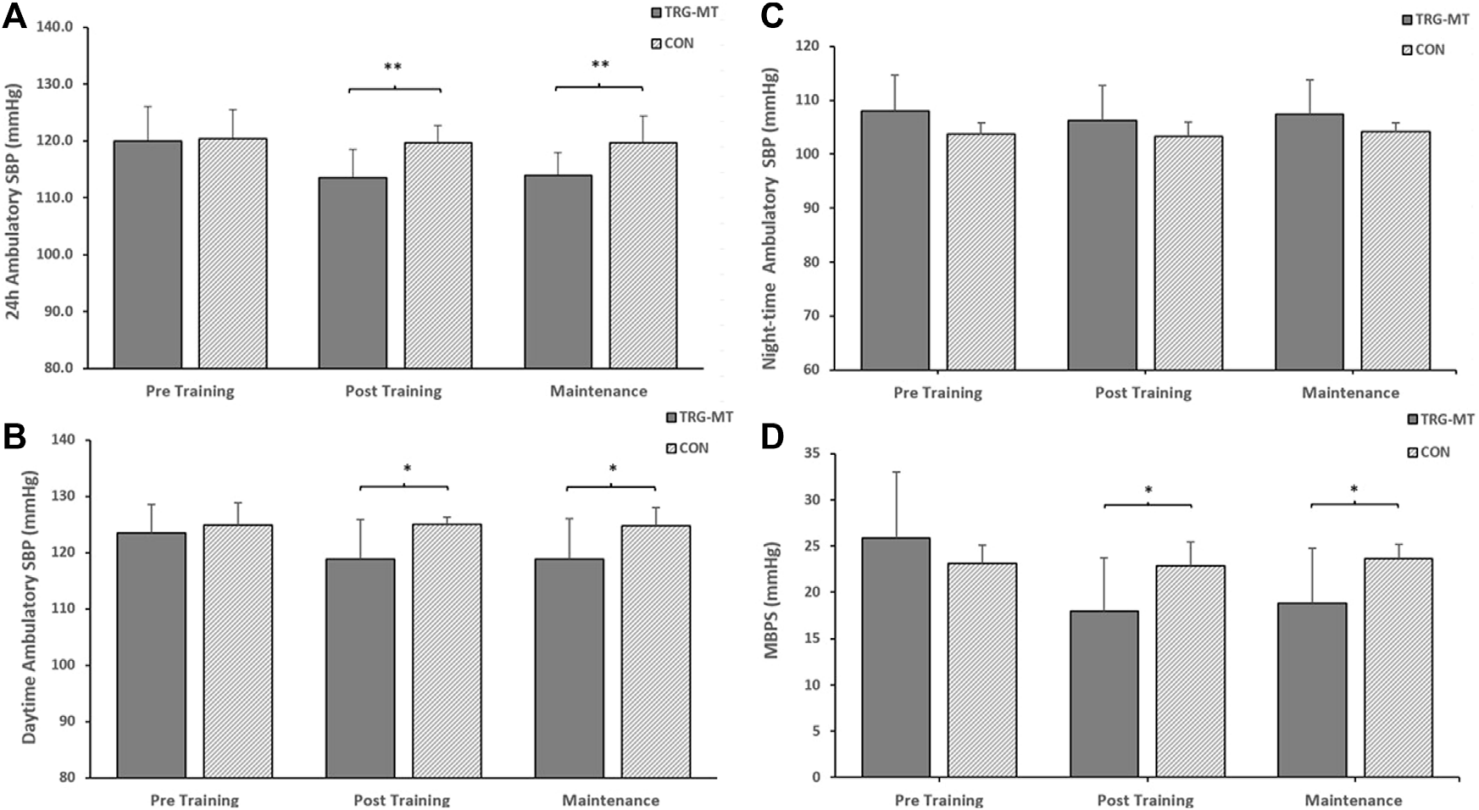
The between-groups effects of 8 weeks of isometric resistance training (IRT) followed by 8 weeks of detraining on **(A)**, 24-h **(B)**, Daytime **(C)**, Night-time ambulatory systolic blood pressure (SBP) and **(D)**, morning blood pressure surge (MBPS) for isometric training-detraining (TRG-DT) and control (CON) groups. *p-value <0.05, **p-value < 0.01.

Following the completion of the 8-week training intervention, the between group (TG-MT and CON) data showed significant differences in 24-h ambulatory SBP (114 ± 7 to 120 ± 3 mmHg, *p <* 0.001), daytime SBP (119 ± 7 to 125 ± 1 mmHg, *p* = 0.016), MPBS (17 ± 7 to 23 ± 5 mmHg, *p* = 0.029) and 24-h SBP ARV (8.20 ± 1.51 to 10.45 ± 1.80 mmHg, *p* = 0.007), daytime SBP ARV (8.43 ± 2.07 to 10.040 ± 1.79 mmHg, *p* = 0.011), with no corresponding significant differences in night-time SBP (106 ± 7 to 103 ± 3 mmHg, *p* = 0.743) or night-time SBP ARV (8.17 ± 3.17 to 8.24 ± 2.78 mmHg, *p* = 0.937). Following the maintenance period these group differences were maintained (24-h, 114 ± 4 to 120 ± 5 mmHg, *p* = 0.006; daytime, 119 ± 7 to 125 ± 3 mmHg, *p* = 0.032; MBPS, 18 ± 7 to 24 ± 2 mmHg, *p* = 0.03; 24-h SBP ARV, 8.26 ± 1.07 to 10.39 ± 2.21 mmHg, *p* = 0.007; daytime SBP ARV, 8.72 ± 1.16 to 10.67 ± 2.56 mmHg, *p* = 0.032; see Figures 3–4). The data between group analysis also confirmed that there were no significant differences in ambulatory DBP or DBP ARV at the end of the IRT programme (mid-point) or following the detraining period (*p* > 0.05 in both cases).

## Discussion

This study is the first to demonstrate that following an 8-week IRT intervention that clinically reduced ambulatory BP (24-h SBP and daytime SBP) and BP diurnal variations (MBPS, 24-h SBP ARV and daytime SBP AVR) in young normotensives, a reduction of the training frequency from three to one session (4 × 2 min contractions with 2 min recovery between contractions) per week is sufficient to maintain these significant reductions up to 8 weeks compared to baseline measures. These reductions in ambulatory BP (24-h, daytime) in addition to those in diurnal variation (MPBS, 24-h SBP ARV, daytime SBP ARV) are in agreement with previous research (Baross et al., 2022b), reinforcing the use of IRT as a therapeutic exercise intervention for the chronic management and possible prevention of hypertension in young and older populations (Wiles et al., 2010; Somani et al., 2018; Baross et al., 2022a).

Comparable reductions in the reported ambulatory BP measures (24-h SBP 7 mmHg; daytime SBP, 4 mmHg) have been seen in previous normotensive IRT studies (24-h SBP, 4 mmHg; daytime SBP, 3–5 mmHg; Somani et al., 2017; Baross et al., 2022a; Baross et al., 2022b), as have the nonsignificant changes in night-time ambulatory SBP (Stiller-Moldovan et al., 2012; Baross et al., 2022a; Baross et al., 2022b), although others (Somani et al., 2017; Taylor et al., 2019) have reported significant reductions (4–6 mmHg). The significant reductions in MBPS (8 mmHg) are again similar to previous works (7 mmHg; Baross et al., 2022a; 6 mmHg; Baross et al., 2022b) as are the reported significant reductions in 24-h SBP ARV (2.1 mmHg) and daytime SBP ARV (2.3 mmHg) in normotensive individuals (24-h 2.216 mmHg, daytime 1.985 mmHg; Baross et al., 2022b), nor does the data differ to those reported in borderline hypertensive (24-h; 2.33 mmHg, daytime 2.45 mmHg; Taylor et al., 2019) populations. Collectively, these findings add further weight to the efficacy of IRT to reduce BP in a range of populations with implications for exercise prescription in a range of exercise-intolerant populations and individuals that have difficulty maintaining a pharmacological strategy.

Despite the abundance of research supporting the use of exercise programmes (aerobic and resistance training) as therapeutic interventions for reducing BP (Saco-Ledo et al., 2020; Lopes et al., 2021) and consequently, the burden placed upon the health services, the adherence, compliance and continued engagement following the completion of the programme appears to be low (Saida et al., 2017; Lopes et al., 2021), with reports of more than half of patients enrolled on a therapeutic exercise intervention failing to engage in the exercise within the first 12 months (Resurrección et al., 2017; Saida et al., 2017). Although it is not fully understood, a “lack of time” (Gee et al., 2012) is cited as one of the main barriers to exercise adherence and compliance, ergo developing a time efficient, efficacious therapeutic intervention should be a healthcare priority, which may be achieved *via* home-based strategies or reduced training sessions. Potentially of greater importance in the present study is the novel finding that the decreases seen in ambulatory BP following 8 weeks of IRT in young healthy normotensives can be maintained over an 8-week maintenance period when IRT is reduced to one bout of 4 × 2 min contractions at 20% MVC, with 2 min recovery between contractions. Thus, a minimal dosage of one session of IRT per week is sufficient to maintain these reductions, a finding with important implications for long-term clinical exercise prescription.

Maintenance dose studies are seldom examined within the exercise medicine literature, however, of the limited work undertaken, time availability is considered to be a key determinant of adherence. Therefore, reducing the time demands of the therapeutic exercise intervention once the desired outcome has been achieved may elicit an increase in the continued engagement with the prescribed exercise to further enhance or sustain training-induced adaptations. It is therefore important to identify the possibility of a reduced dose of exercise, in this case the number of weekly sessions once the therapeutic exercise intervention has provided the required reductions in BP, with the emphasis of the intervention shifting to maintaining these reductions which may improve individual’s adherence and compliance.

Previous studies undertaking aerobic and resistance training interventions have reported similar finding to those presented here. Hickson and Rosenkoetter (1981) demonstrated that aerobic capacity was maintained when training frequency was reduced (6–2 sessions per week) for a 15-week period. As with the present study the exercise session volume and intensity were maintained. Further, Hickson et al. (1985) reported short term aerobic capacity was maintained for a 15-week period following an equivalent reduction in training volume to the present study (66% reduction) in aerobic training volume, again similar to the present study (24 min–8 min, 66% reduction), for a 15-week period with no changes to the exercise intensity, reported participants maintained short term aerobic capacity. Additionally, resistance training interventions such as Bickel et al. (2011) have reported a reduced training frequency from thrice to once weekly following a 16-week resistance training period was sufficient to further increase myofiber hypertrophy. More recently, continued strength improvements have been noted following a once-a-week maintenance resistance training period preceded by a 20-week (3 sessions per week) training programme (Antunes et al., 2022). More specifically Cohen et al. (2023) employed a similar intervention to the present study, demonstrating that a single isometric training bout (4 × 2-min wall squats) was not only sufficient in maintaining significant reductions in resting SBP obtained upon completion of the IRT programme, but elicited further decrease (1.8 mmHg) in resting BP following the fulfilment of the maintenance dose. Collectively, these findings provide further support for the implementation of a maintenance dose once the initial therapeutic benefits of the intervention have been achieved, which could overcome self-reported barriers to exercise, thus increasing the effectiveness of IRT as a non-pharmacological strategy to counteract hypertension.

One limitation to the present study is that the participants were normotensive and thus, these findings cannot be generalised to those that display elevated BP, therefore we recommend that future studies should investigate if these findings are replicated in hypertensive cohorts. Although participant numbers were determined using an *a priori* sample size calculations we acknowledge that the group numbers are low, possibly compromising our confidence in the findings and may explain the lack of change in ambulatory night-time SBP previously seen in some studies. Additionally, as adherence and compliance to the training have been reported to decrease over a short period of time, with “lack of time” to undertake the therapeutic exercise intervention cited as the main reason, it is important to examine if a reduced training volume will maintain the reductions in BP when unsupervised and therefore, maintain the clinical benefits.

## Conclusion

The novel data presented here demonstrates that the observed reductions in both ambulatory BP (24-h SBP and daytime SBP), in addition to BP diurnal variations (MBPS, 24-h SBP ARV and daytime SBP AVR) can be maintained following an 8-week maintenance dose consisting of one bout of 4 × 2 min isometric knee extensor contractions in normotensives. The data add further weight to the growing body of evidence promoting IRT as an effective therapeutic exercise intervention to reduce BP. Furthermore, the data indicate that IRT as a therapeutic intervention may have an important role in the chronic management of BP and could be pivotal to reducing the global prevalence of hypertension.

## Data Availability

The datasets presented in this study can be found in online repositories. The names of the repository/repositories and accession number(s) can be found below: https://doi.org/10.24339/c8613cc2-1757-4063-bebe-f1f263fc1956.
