# Supplementary material for: Effects of a maintenance period on ambulatory blood pressure and morning blood pressure surge in young normotensives post isometric training
Source: Front Physiol. 2024 Aug 15;15:1405230. doi: 10.3389/fphys.2024.1405230 (PMC11358553; doi:10.3389/fphys.2024.1405230)
Supplement: Supplementary file 1 [file Table1.DOCX]

**Table 1. Participant baseline demographic and ambulatory data**

TG-MT group (n = 13) CON group (n = 9)

Age (yrs) 21 ± 2 23 ± 3

Height (cm) 169 ± 9 170 ± 10

Body Mass (kg) 73 ± 18 74 ± 8

Resting Heart Rate 69 ± 10 69 ± 9

***Ambulatory BP***

24-hour SBP (mmHg) 120 ± 6 121 ± 5

24-hour DBP (mmHg) 64 ± 6 64 ± 6

Daytime SBP (mmHg) 123 ± 5 125 ± 6

Daytime DBP (mmHg) 71 ± 6 66 ± 8

Night-time SBP (mmHg) 108 ± 7 105 ± 3

Night-time DBP (mmHg) 55 ± 8 56 ± 7

Morning SBP (mmHg) 122 ± 7 120 ± 4

Lowest Night-time SBP (mmHg) 96 ± 8 97 ± 4

MBPS (mmHg) 26 ± 7 23 ± 6

Values are means ± SD. TG-MT, maintenance group; CON control group; SBP, systolic blood pressure; DBP, diastolic blood pressure; MAP, mean arterial pressure; MBPS, morning blood pressure surge.
