# Supplementary material for: Effects of a maintenance period on ambulatory blood pressure and morning blood pressure surge in young normotensives post isometric training
Source: Front Physiol. 2024 Aug 15;15:1405230. doi: 10.3389/fphys.2024.1405230 (PMC11358553; doi:10.3389/fphys.2024.1405230)
Supplement: Supplementary file 2 [file Table2.DOCX]

**Table 2. Ambulatory SBP ARV results at baseline, post 8-week IRT and post 8-week maintenance period**

TRG-MT Control

| **Ambulatory SBP ARV**  **(mmHg)** | **Baseline** | **Post 8-week IRT** | **Post 8-week maintenance** | **Baseline** | **Post 8-week IRT** | **Post 8-week maintenance** |
| --- | --- | --- | --- | --- | --- | --- |
| **24-h** | 10.29 ± 1.72 | 8.20 ± 1.51* | 8.26 ± 1.05** | 10.12 ± 1.78 | 10.45 ± 1.80 | 10.39 ± 2.21 |
| **Daytime** | 10.77 ± 2.07 | 8.45 ± 2.07** | 8.72 ± 1.16** | 10.45 ± 2.40 | 10.04 ± 1.79 | 10.67 ± 2.56 |
| **Night-time** | 8.92 ± 2.84 | 8.17 ± 3.17 | 8.27 ± 1.68 | 8.11 ± 1.97 | 8.24 ± 2.78 | 8.61 ± 1.94 |

Data are presented as mean ± SD. (TRG-MT group n=13; Control group n=12). SBP, systolic blood pressure; ARV, average real variability. P values represent changes within groups over time compared to baseline measures. ** P value < 0.01, * P value < 0.05.
